# Phytoniosome: a Novel Drug Delivery for Myrtle Extract

**Published:** 2018

**Authors:** Mahboobeh Raeiszadeh, Abbas Pardakhty, Fariba Sharififar, Mehrnaz Mehrabani, Hojjat Nejat-mehrab-kermani, Mitra Mehrabani

**Affiliations:** a *Herbal and Traditional Medicines Research Center, Kerman University of Medical Sciences, Kerman, Iran. *; b *Department of Traditional pharmacy, School of persian Medicine, Kerman University of Medical Sciences, Kerman, Iran. *; c *Pharmaceutics Research Center, Neuropharmacology Institute, Kerman University of Medical Sciences, Kerman, Iran.*; d *Physiology Research Center, Institute of Basic and Clinical Physiology Sciences, Kerman University of Medical Sciences, Kerman, Iran. *; e *Department of Microbiology and Virology, School of Medicine, Kerman University of Medical Sciences, Kerman, Iran.*

**Keywords:** Encapsulation efficiency, *Myrtus communis*, Myrtle, Phytoniosome, Release, Stability

## Abstract

Traditionally, *Myrtus communis* (myrtle) has been used for treatment of several kinds of disorders. However, there are some factors, namely, low solubility and permeability, which restrict use of myrtle extract (ME) in medical applications. Regarding these limitations, the aim of the present study was to develop a new niosomal formulation to enhance ME stability and permeability. Briefly, several niosomal formulations were prepared by non-ionic surfactants and cholesterol with different molar ratios. Afterward, size, entrapment efficiency (EE%), release and stability of niosomal myrtle extract (nME) were investigated. The effect of ME and nME on viability of 3T3 cells was evaluated using MTT assay. Antibacterial activity of ME and nME was also assessed against *Staphylococcus aureus*, *Staphylococcus epidermidis*, *Escherichia coli, Micrococcus luteus, and Bacillus subtilis*. Sizes of niosomes were 5.3 ± 0.3 to 15.9 ± 2.2 µm with 4.1 ± 0.3 to 26.9 ± 1.7 mV zeta potential. The EE% of niosomes was varied from 45.4% to 93.4%. An *in-vitro *release study on F5 formulation (Span60: Tween60: cholesterol (3:3:4 molar ratio)) revealed that about 36.9%, 38.5% and 26.7% of phytoconstituents were released within 12 h from acetate cellulose membrane, 0.45 µm, regenerated cellulose membrane, 0.45 µm, and cellophane dialysis sack, 12000 Da, respectively. F5 formulation significantly showed lower toxicity on cells. It had higher antibacterial activity that has been shown by lower MICs and higher zone of inhibition compared to ME.

Overall, F5 formulation in the presence of 4% ME produced stable multi lamellar vesicles with optimal *in-vitro *release and EE%. This formulation also exhibited better antibacterial activity than ME.

## Introduction

For thousands of years, herbal medicines have been used to treat a variety of diseases particularly skin disorders such as dermatitis, burn, and wound (1). Among plant components, water soluble ones including flavonoids, tannins and terpenoids have been reported to have significant therapeutic effects. However, due to their large molecular size and poor lipid solubility, polyphenolic molecules have low bioavailability, are poorly absorbed and are reached at low concentration to sites of action (2). Therefore, using new drug delivery systems like liposomes or niosomes are helpful in topical herbal formulations (3-4).

Niosomes are composed of non-ionic surfactants which form a spherical-shaped bilayer membrane (5).Cholesterol (Chol) and charge-inducer molecules are oftentimes utilized in the niosome preparation. The former acts as a stabilizing agent for bilayer membrane while the latter produces repulsive force by developing a charge on the surface of niosomes, thereby stabilizing and preventing aggregation of the prepared formulation (6). Niosomes are very similar to liposomes, but they are composed of synthetic non-ionic surfactants, e.g. sorbitans esters (Spans) or ethoxylated esters (Tweens) instead of phospholipids that are more vulnerable to heat and oxidation-mediated degradation (7). The greater stability, lower cost, and more importantly biodegradable, biocompatible, and nonimmunogenic properties have led to the exploitation of these drug delivery systems as alternatives to liposomes (8-9). In topical dosage forms, niosomes are used to decrease and modify the release of active drug, resulting in a sustained release profile, drug targeting and less toxicity (10).

Niosomes similar to other drug delivery systems have been used in gene delivery systems (11). Also the phytosome technology was successfully applied to encapsulate herbal extracts (ginkgo, milk thistle, green tea, marigold) as well as phytochemicals (curcumin, silybinin) yielding remarkable results both in *in-vivo* and *in-vitro* pharmacokinetic studies (2, 12-15). Manosroi’s *et al*. results have shown that rice bran bioactive compounds or gallic acid niosomal formulations exhibited an acceptable antiaging activity and a higher chemical stability, allowing it to be used as a novel drug delivery systems (16-17). A similar study indicated that niosomes are promising carriers for topical delivery of caffeine and gallic acid compared with black tea extract in skin care products (18). 

A well-known medicinal plant used worldwide in traditional medicine,* M. communis* L. is part of botanical family of Myrtaceae. In traditional Persian books, it was called “Ace” or “Amar”, which means good and aromatic plant. It is an evergreen, bushy tree often found in Mediterranean countries, Asia, America, Southern Russia, New Zealand and some other countries in the World (19-20). In Iranian traditional medicine, the herb has been used topically to treat mouth ulcer, nosebleed, burn, wound, and has been applied as an antiseptic and disinfectant remedy as well (21-22). However, there are some limitations for topical usage of ME resulted from its poor biopharmaceutical properties, low solubility and low permeability (2). So, niosomal formulation is beneficial for myrtle extract (ME) release and bioavailability in topical formulations.

The aim of the present study was to investigate the phytoniosomal dispersion of myrtle’s hydroalcoholic extract using non-ionic surfactants and cholesterol (Chol). The optimized formulations were screened for EE%, release profile, vesicle size and stability studies. Finally, antibacterial and cytotoxicity effect of the selected niosomal myrtle extract (nME) formulation (F5) were evaluated in comparison to those of ME.

## Experimental

*Materials*

*Plant*

The aerial parts of *M. communis* were collected from Haji Abad, Iran during July/August 2015. The sample was identified by a professional herbalist and a voucher specimen (KF 1356) was also prepared and deposited at the herbarium of faculty of Pharmacy, Kerman University of Medical Sciences for future reference. Myrtle leaves were manually isolated from the aerial parts, then were dried in a dry and shady place at ambient temperature for one month. The dried parts were grinded in to coarse powder and kept in an air tight and light-resistant container for future experiments.

*Chemical*

Sorbitane laurate (Span 20), sorbitane monopalmitate (Span 40), sorbitane stearate (Span 60), Sorbitane monooleate (Span 80) and polysorbate 20, 40, 60 and 80, Gallic acid (3,4,5-trihydroxybenzoic acid) and Folin–Ciocalteau reagent, filter membrane containing cellophane 12000 D, acetate cellulose 0.45 µm and regenerated cellulose 0.45 µm were purchased from Sigma Chemical (St. Louis, MO, USA). INT (2-(p-iodophenyl)-3-(p-nitrophenyl)-5phenyl tetrazolium chloride), cholesterol (Chol), Muller- Hinton agar medium, Muller- Hinton broth medium, chloroform, DMSO and ethanol were obtained from Merck Company (Darmstadt, Germany). All other reagents were of an analytical grade.

*Cell Culture*

3T3 mouse embryo fibroblast cell line, obtained from Pasteur Institute of Iran (Tehran, Iran), was routinely grown in DMEM with 10% FBS, and 1% penicillin/ streptomycin at 37 °C in a humidiﬁed atmosphere of 5% CO2.

*Bacteria*

*Staphylococcus aureus* (PTCC 1112), *Escherichia coli* (PTCC 1330), *Micrococcus luteus* (PTCC 1110) *Staphylococcus epidermidis *(PTCC 1114), and *Bacillus subtilis* (PTCC 1023), were obtained from the Department of Microbiology, School of Medicine, Kerman University of Medical Sciences. They were maintained in Muller- Hinton agar slants at 4 °C throughout the study and used as stock 

cultures.

*Methods*

*Extract preparation*

The extraction was prepared by putting 100 g pulverized myrtle leaves into a percolator column, to which then ethanol 80% was continuously added. The solvent flew through the column at 10 mL/h at 25 °C. Total extraction was concentrated in a rotary evaporator at 45 °C and was dried in an oven at 40 °C. Dried ME was stored in sealed vials at –20 °C for further analysis. 

*Determination of total phenol content of the extract*

Total phenolic content in ME was determined spectrophotometrically using the Folin-Ciocalteu reagent assay with gallic acid as standard according to the previously reported method with a slight modification (23).Briefly, 100 μL ME or a standard solution of gallic acid was added to a test tube, then mixed with 500 μL of diluted Folin-Ciocalteu reagent (1:10 v/v) and the resulted mixture was slightly shaken for 2 min. Afterwards, 400 μL of an aqueous solution of Na_2_CO_3_ (7.5% w/v) was added and the obtained mixture was incubated for 30 min in the dark at 25 °C. After incubation, 4000 μL distilled water was added to the test tube and the mixture was centrifuged at 3000 rpm for 2 min so as to separate probable sediment. The absorbance, relative to that of a blank prepared using ethanol 50%, was measured at 765 nm using multi-mode microplate reader (BioTek^®^, USA). 

The linearity of this assay was determined as 150-500 µg/mL gallic acid equivalents. The concentration of total phenolic compounds in the ME was determined as mg of gallic acid/ g total extract by using regression equation that obtained from the calibration curve of the gallic acid standard. All determinations were performed three times.

*Niosome preparation*

Film hydration method was used to prepare niosome (24). Briefly, 1200 µmol of the non ionic surfactants including Span (S) and Tween (T) and Chol in different molar ratios; 3.5:3.5:3, 3:3:4 and 2.5:2.5:5, respectively, were dissolved in chloroform: ethanol with 2:1 ratio in a 1000 mL round-bottom flask. Afterwards, 6.6 mL of ME solution 6% was added to the lipid phase. The organic solvents were removed under vacuum in a rotary evaporator (EYELA SB-1200, Japan) at 50 °C for 30 min to form a thin film on the wall of the flask. Residual solvents were evaporated in a vacuum oven for 12 h at 30 °C. The film was then hydrated with 10 mL deionized water with a gentle rotation in water bath at 50 °C for 60 min to produce an aqueous niosomal suspension containing 4% ME. The phytoniosome suspension was left to mature overnight at room temperature and then stored in refrigerator for further studies.

*Measuring particle size*

The size of phytoniosomes was measured by laser light scattering method in a Malvern particle size analyzer, (Malvern Instruments, Master Sizer X-100, UK) 24 h after preparation for all formulations and also over a period of three months for stability measurements. The fundamental size distribution derived from this technique was volume based (dv). For size measurements, the preparation was appropriately diluted with deionized water and the obscuration level was kept at 16% at a stable count rate. The values for the particle mean size diameters of different formulations were compared to each other.

*Optical microscopy*

Optical microscopy (Leitz, HM-LUX3, Germany) was performed to illustrate the number and morphological differences between formulated phytoniosomes prepared with different surfactants and different molar ratio. 

*Scanning electron microscopy (SEM)*

Scanning electron microscopy was used for surface morphologic and topographic evaluation of developed niosomal formulation. After preparing the sample, the size and morphology were observed at 30 kV using a KYKY- EM3200 electron microscope.

*Determining the zeta potential value*

The zeta potential values of phytoniosomes were obtained through high resolution Laser Doppler Electrophoretic technique using WALLIS zeta potential analyzer (Corduan, France).

*Encapsulation efficiency in vesicles*

To separate the non-entrapped drug, the vesicle suspensions were centrifuged (Vision/ VS-35SMTi, Korea) at 59000 × g for 30 min at 6 °C. After that the supernatant phase was separated and the pellets were disrupted by isopropyl alcohol. Then the amount of active constituent in the supernatant and also in the pellets was determined by Follin-Ciocalteu assay. All the analyses were carried out in triplicate and the values were averaged. EE% was calculated as follows:

EE% = 100 × amount of entrapped drug/ amount of drug used for vesicle preparation

*Studying vesicle stability*

The stability of the selected formulations was assessed in terms of size, constituent separation, as well as EE%. According to the International Conference on Harmonization of Technical Requirements for Registration of Pharmaceuticals for Human Use (ICH) guidelines, Iran is categorized in zone II. As far as accelerated and intermediate testing condition is concerned, formulations were stored under three conditions such as 4 °C, 25 °C with relative humidity (RH) of 30%, and 40 °C with RH of 70%. Then, phytoniosomes were examined during 24 h, 2 weeks, 1 and 3 months after preparation. No special precautions were taken to improve the stability of vesicles.

*Phytoconstituents release evaluation*

The *in-vitro* release study was performed using static vertical diffusion Franz cells with an effective diffusion area of 1.5 cm^2^ and a receptor phase volume of 15 mL (Ashke-shisheh Co., Iran). The acetate cellulose dialysis sack (cut-off 12000 Da), regenerated cellulose membrane (0.45 µm) and acetate cellulose membrane (0.45 µm) were soaked in ethanol 50% for 24 h before the experiments. The membrane was fixed between donor and receptor compartments. The receptor compartment was filled with acceptor phase which contains 50% ethanol 98° and 50% distilled water, then it was continuously stirred and thermostated at 37 ± 1 °C throughout the experiment and also the donor compartment was filled with 1 mL phytoniosome (25). ME solution and empty niosomal formulation were used as control. One mL sample was withdrawn at fixed time intervals from receptor compartment and replaced with an equal volume of fresh acceptor phase to ensure sink conditions. The permeated drugs concentrations were measured by Folin-Ciocalteu method like EE%.

*MTT Reduction Assay*

MTT assay is an established colorimetric method for determining the viability of cells in cytotoxicity and proliferation studies. 3T3 Cells were seeded in 96-well plates at density of 5000 cells per well. After 24 h, they were exposed to different concentrations of ME and F5 formulation for 24 h. MTT was added to each well and the cells were incubated for 4 h in a humid incubator at 37 °C. Afterwards, the medium was removed and 100 μL of dimethyl sulfoxide (DMSO) was added. Absorbance was measured at 570 nm by a microplate reader (BioTek ELX800, Winooski, Vermont, USA).

*Antibacterial activity*

*Determining minimum inhibitory concentrations (MICs)*

MICs for ME and F5 formulation were determined by micro broth dilution method. In this method a series of two-folded dilutions ranging 16 to 0.125 mg/mL were prepared in Muller- Hinton broth with 0·5% (v/v) Tween 80 to enhance solubility. Inoculates were prepared by diluting an overnight culture of the pathogen in 0.9% NaCl solution and turbidity was adjusted to 0.5 McFarland standard. 100 µL of inoculum containing 10^5 ^CFU/mL bacteria and 100 µL of dilutions were added to wells and incubated at 37 °C for 24 h. After that 20 µL of INT solution 0.5% (w/v) was added to wells. After 30 min incubation at 37 °C, the pink color in wells indicated the microbial growth. Finally the lowest concentration inhibiting bacterial growth was reported as the MIC.

**Table 1:** Organoleptic and physicochemical evaluation of myrtle leaves.

**Parameters**	**Value**
Color	Green
Odor	Aromatic
Taste	Bitter and intensive
Shape	Lanceolate
Total ash	4.53±0.18 %
Water soluble ash	4.32±0.21 %
Acid insoluble ash	0.18±0.01 %
Loss on drying	5.54±0.23 %
Extractive value	31.78±1.05 %w/w
Essential oil value	0.94±0.01 %v/w

**Table 2:** Phytochemical analysis of myrtle ethanolic extract.

**Phytochemical constituent**	**Test applied**	**Result**
Alkaloids	Dragendroffs and mayer reagent	-
Tannin	Ferric chloride test solution	+
Flavonoid	Lead acetate and dilute ammonia test	+
Saponin	Froth test	-
Anthraquinone	Borntragers test	-
Cardiac glycoside	Keller killianis test	-
Steroid	Liebermannburchard test	-
Terpenoid	Salkowskis test	+

**Table 3:** Effect of surfactants and cholesterol on size, zeta potential and EE% of phytoniosomes.

**Formulation** ** ID**	**Surfactants molar ratio**					**Size (µm) ± SD**	** Zeta potential ** **(mV) ± SD**	**EE% ± SD**
	**Span 40**	**Tween 40**	**Span 60**	**Tween 60**	**Cholesterol**			
F1	3.5	3.5	˗	˗	3	6.19±0.43	26.39±1.08	45.4±3.8
F2	3	3	˗	˗	4	5.59±0.37	26.95±1.76	67.4±4.3
F3	2.5	2.5	˗	˗	5	15.87±2.27	4.15±0.33	60.1±4.1
F4	˗	˗	3.5	3.5	3	5.28±0.31	25.33±1.40	90.8±4.6
F5	˗	˗	3	3	4	7.29±0.47	25.66±2.19	91.5±5.3
F6	˗	˗	2.5	2.5	5	8.60±0.60	24.52±1.98	93.4±3.3

**EE%** entrapment efficiency percent.

**Table 4:** Effect of storage on change in constituent separation, size and EE% of phytoniosomes at different time intervals after preparation.

**ID**	**Storage condition**	**Constituent separation**			**Mean volume diameter (µm) ± SD**			**EE% ± SD**		
		**2 weeks**	**1 month**	**3 months**	**2 weeks**	**1 month**	**3 months**	**2 weeks**	**1 month**	**3 months**
F1	I	N	N	N	6.04±0.43	6.36±0.58	7.60±0.81	46.9±2.4	57.0±2.1	58.4±3.3
	II	P	P	P	5.79±0.25	6.58±0.79	24.52±5.14	54.2±2.7	59.7±3.2	62.0±3.7
	III	P	P	P	6.40±0.30	12.11±1.35	13.68±3.21	49.0±3.5	60.9±2.2	62.3±3.1
F2	I	N	N	N	6.67±0.55	6.94±0.46	8.09±1.17	65.7±2.6	76.5±4.2	74.7±3.7
	II	N	P	P	6.20±0.30	6.57±0.74	8.02±1.08	67.4±2.1	71.9±3.9	76.1±1.0
	III	P	P	P	6.67±0.56	8.43±0.98	69.92±9.94	67.3±3.1	79.7±4.1	82.6±4.3
F4	I	N	N	N	7.19±0.86	7.97±1.72	8.00±0.79	74.8±2.2	80.4±2.0	86.1±3.4
	II	N	P	P	6.98±0.52	7.94±0.57	8.64±1.12	87.7±3.1	88.5±2.9	83.8±4.4
	III	P	P	P	8.48±1.48	9.79±2.04	34.39±12.96	65.5±2.9	66.8±2.3	76.1±4.9
F5	I	N	N	N	7.23±0.24	7.59±0.56	7.77±0.46	92.2±3.4	95.0±2.3	95.8±4.4
	II	N	P	P	7.20±0.46	7.68±0.36	8.89±0.71	88.7±3.7	91.2±2.9	92.3±2.7
	III	N	P	P	7.62±0.25	8.72±0.64	9.85±0.98	87.7±2.5	87.9±3.5	91.1±3.4
F6	I	N	N	N	8.96±2.46	8.50±0.62	9.77±1.69	95.1±3.2	97.6±3.1	97.8±4.2
	II	N	P	P	7.98±0.96	8.90±1.24	9.66±1.09	94.4±4.2	92.6±5.3	93.4±4.7
	III	P	P	P	7.50±0.62	9.80±1.48	13.67±2.88	93.5±3.1	93.9±3.8	97.1±4.2

**EE%** entrapment efficiency percent.

F1; S40: T40: Chol (3.5:3.5:3 molar ratio)

F2; S40: T40: Chol (3:3:4 molar ratio)

F4; S60: T60: Chol (3.5:3.5:3 molar ratio)

F5; S60: T60: Chol (3:3:4 molar ratio)

F6; S60: T60: Chol (2.5:2.5:5 molar ratio)

I; 4 °C, II; 25 °C , 30% RH, III; 40 °C, 70% RH

N; normal, *P*; precipitate

**Table 5:** Release properties of ME and F5 formulation.

**Formulation**	**Membrane**	**Accumulative release %**	**Release model**	**R** ^2^ ** value**
ME	Acetate cellulose 0.45 µm	70.4	Peppas equation	1
	Regenerated cellulose 0.45 µm	31.0	Peppas equation	0.828
	Cellophane 12000 D	74.6	Zero order equation	0.997
F5	Acetate cellulose 0.45 µm	36.9	Peppas equation	0.909
	Regenerated cellulose 0.45 µm	38.5	Peppas equation	0.942
	Cellophane 12000 D	26.7	Higuchi equation	0.97

**ME**; myrtle extract

**F5**; S60: T60: Chol (3:3:4 molar ratio)

**Table 6:** Diameter of zone of inhibition and MIC against bacteria by ME and F5 formulation

**Microorganisms**		***S. aureus***	***S. epidermidis***	***M. luteus***	***B. subtilis***	***E. Coli***
**Formulations**						
		**MIC (mg/mL)**				
**ME**		0.125	1	4	0.125	4
**F5**		0.125	0.5	2	0.125	4
**Concentrations (mg/mL)**		**Zone of inhibition (mm)**				
**ME**	8	29.1±1.2	18.7±0.8	13.3±0.9	24.5±1.0	13.1±0.8
	4	23.6±0.9	14.8±0.9	12.3±0.7	20.1±0.9	10.9±0.4
	2	19.2±0.5	11.3±1.1	NA	17.5±0.5	NA
	1	18.2±0.7	10.5±0.9	NA	13.3±0.9	NA
	0.5	14.9±1.2	9.7±0.6	NA	12.9±0.5	NA
**F5**	8	35.1±1.5***	25.4±1.4***	17.8±0.8***	30.5±1.3***	12.4±0.9
	4	28.2±1.1***	18.6±0.4***	16.1±1.0***	22.4±0.9	10.1±0.6
	2	22.6±0.9*	16.0±0.6***	13.5±0.7	17.2±1.1	NA
	1	16.3±0.5	13.5±0.5**	NA	13.4±0.7	NA
	0.5	14.5±0.8	11.7±0.6	NA	12.6±0.5	NA
**Empty niosome**	-	NA	NA	NA	NA	NA
**Gentamicin**	0.01	30.5 ± 0.4	25.1 ± 0.3	30.1 ± 0.4	34.4 ± 0.8	38.3 ± 0.6
**Ciprofloxacin**	0.005	22.2 ± 0.5	36.1 ± 0.2	26.4 ± 0.7	25.3 ± 0.6	20.2 ± 0.3

**ME**; myrtle extract

**F5**; S60: T60: Chol (3:3:4 molar ratio)

**NA**; no activity

*** ; *P *< 0.001 compared to ME

** ; *P *< 0.01 compared to ME

* ; *P *< 0.05 compared to ME

**Figure 1:**
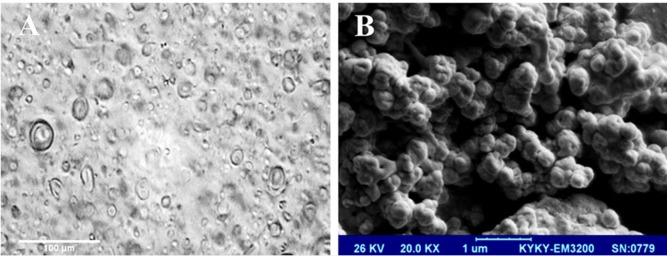
Photomicrographs of F5 formulation (S60: T60: Chol (3:3:4 molar ratio)) (A) optical microscope, (B) scanning electron microscope (SEM). Vesicles are spherical in shape and exist in disperse and aggregate collections. Seen under (A) 400× and (B) 20000× magnification

**Figure 2:**
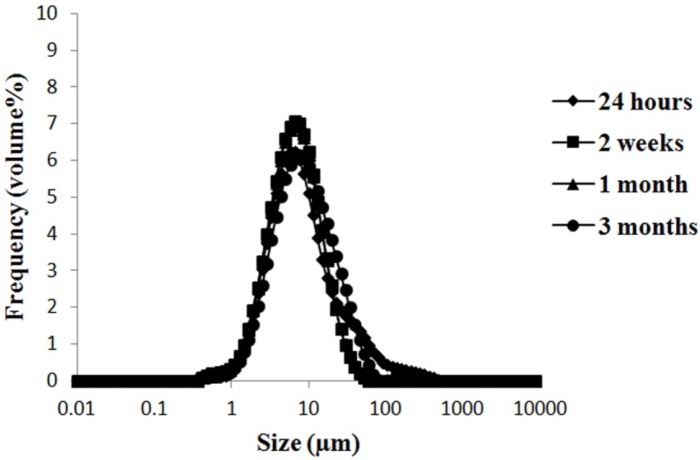
The size distribution changes of F5 formulation (S60: T60: Chol (3:3:4 molar ratio)) during storage at 4 °C as an indicator of physical stability

**Figure 3:**
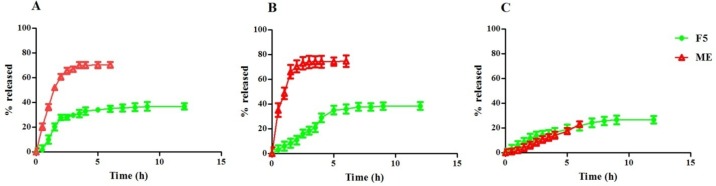
Release profile of ME and F5 formulation (S60: T60: Chol (3:3:4 molar ratio)) from acetate cellulose membrane, 0.45 µm (A), regenerated cellulose membrane, 0.45 µm (B) and cellophane membrane 12000 D (C) in ethanol 50% at 37 °C (mean ± SD, n = 3).

**Figure 4:**
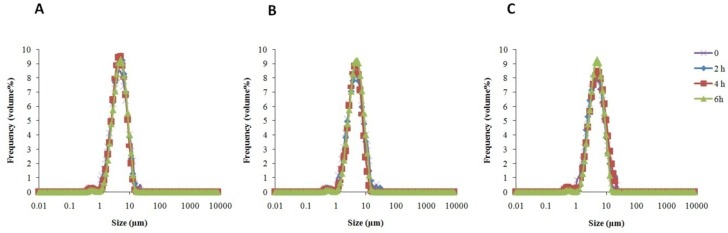
Physical stability of F5 formulation (S60: T60: Chol (3:3:4 molar ratio)) during release process from acetate cellulose membrane, 0.45 µm (A), regenerated cellulose membrane, 0.45 µm (B) and cellophane membrane 12000 D (C) in ethanol 50% at 37 °C (mean ± SD, n = 3).

**Figure 5:**
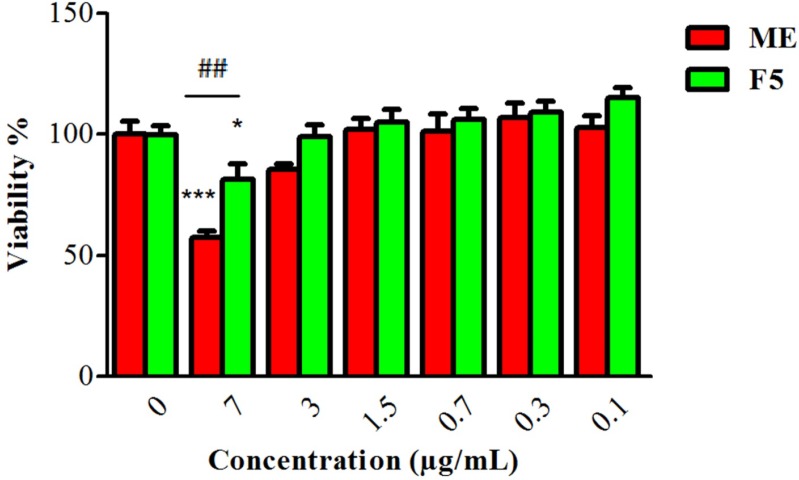
Cytotoxic effects of ME (myrtle extract) and F5 formulation (S60: T60: Chol 3:3:4) on 3T3 cells following 24 h incubation (n = 3; mean ± SD).

*Disc diffusion method*

To screen the antimicrobial activity of ME and F5 formulation, the disc diffusion method was carried out. Inoculate containing 10^5 ^CFU/mL bacteria was used to uniformly lawn Muller Hinton agar plates using a sterile cotton swab in order to get an identical microbial growth on plates. The two-folded dilutions ranging 8 to 0.5 mg/mL were prepared. Under aseptic conditions, empty sterilized discs (6.4 mm) were impregnated with 100 μL of different concentrations and placed on the agar surface with equidistance to each other. Disc moistened with 2% DMSO or empty niosome were used as vehicle control and blank, respectively. Also antibiotic discs including ciprofloxacin (5 µg/mL) and gentamicin (10 µg/mL) were used as positive controls. After 24 h of incubation at 37 °C, the zone of inhibition was measured with a ruler.

*Statistical analysis*

Results were expressed as mean ± standard deviation. Moreover, statistical analysis was performed using one-way ANOVA, followed by Tukey′s post hoc test in SPSS software. *P *values lower than 0.05 was considered as significant.

## Results and Discussion

*Phytochemical analysis and measurement the amount of total phenolic content*

The results of organoleptic and physicochemical screening of myrtle leaves are given in Table 1.

Phytochemical analysis was undertaken using standard qualitative methods. Likewise, Table 2 reflects that ME has tannin, flavonoid, and terpenoid. 

The total phenolic content was determined by the Folin-Ciocalteau method. The results from regression equation of the calibration curve (y = 0.0012x+0.0175, R^2 ^= 0.999) were determined as gallic acid (GAE) equivalents per 1.0 g of ME (GAE/g extract). According to the findings, the phenolic content of ethanolic ME was equivalent to 264.86 ± 4.19 mg GAE. Gradeli *et al*. reported that the leaf methanolic extract of Greece myrtle have 373 mg GAE/g extract. Moreover, they showed that the phenolic content is dependent on harvest seasons. According to their results, the highest accumulation of phenolic constituents occurred in August when it is the full flowering stage for myrtle (26).

*Vesicle forming ability of surfactants*

To investigate the influence of surfactant structure on EE% of ME, phytoniosomal formulations of different Spans and Tweens were prepared. In the presence of S20 and S80 surfactants, vesicle yield was low along with many separated crystals. Results listed in Table 3 show that S60/T60 formulations have higher EE% than vesicles prepared by S40/T40 (*P* < 0.05). As shown in similar studies, the surfactant’s chemical structure positively influences the EE% due to increase in the alkyl chain length of surfactants (27-28). The EE% followed the trend S60 (C_18_) > S40 (C_16_) > S20 (C_12_) > S80 (C_18_) (29).The reason why vesicles are not formed in the presence of plant extract can possibly be due to the short alkyl chain of S20 (lauryl, C12). S60 and S80 have the same head groups whereas S80 has an unsaturated alkyl chain and there are some reports about the instability of its niosomal formulations (30). Phytoniosomes almost at one main form, multi-lamellar vesicles (MLVs), were obtained from the S40/T40 and S60/T60 surfactants in the presence of Chol with different molar ratio.

*Effect of cholesterol content*

Chol is the most common additive in the niosomal systems, influencing vesicle stability and permeability (27, 31). The effect of Chol on ME entrapment was varied according to the nonionic surfactant used. In the present study, the influence of added Chol on EE% was evaluated by changing Chol molar ratio from 30% to 50%. It was found that in S60/T60 formulation, the EE% was coordinately increased with Chol molar ratio. As shown in a previous study, when the hydrophilic-lipophilic balance (HLB) of the surfactant increased above 10, the amount of needed Chol increased to form vesicles (32). We found that a molar ratio of 4:3:3 between Chol, S, and T is an optimal ratio for the formulation of physically stable nME.

*Effect of extract concentration*

As other researches show, most of the myrtle topical formulations contain 3-5% extract (33-35). Babaee *et al*. have shown that a paste containing myrtle (5%) could enhance treatment efficacy of recurrent aphthous stomatitis (RAS) over placebo paste (33). Similar investigations have revealed that topical essential oil (5%) and topical decoction (5%) of *M. communis *decreased the mean time of pain relief and attenuated the size of ulcers in patients with minor RAS (34). Camargo *et al*. have demonstrated that 3% *M. communis *hydrolyzed extract enhanced skin hydration by topical usage (35). So, in this study, the phytoniosomes of 2, 3, 4, and 5% w/v ME were prepared using the 1200 µmol amount of Chol and surfactants. The results revealed that the amount of ME higher than 4% was not appropriate to form stable phytoniosomes, because of ME sedimentation in formulations.

*Particle size*

An important physical characteristic of the vesicular drug delivery systems is the vesicle size, which may be determined by different techniques. In the present study, the mean volume diameter of phytoniosomes was evaluated by laser light scattering technique. HLB of used surfactants and Chol content are two parameters which effected particle size. Chol is able to induce vesicle formation in different types of surfactants. As Chol increases the chain order and stabilizes the bilayers of vesicles, it is expected that vesicles with relatively high Chol content can be smaller than vesicles with low amounts of Chol. Nonetheless, changing the amount of Chol could have obvious effects on the niosomal mean diameter as shown in several scientific studies (36-37). Generally, by increasing the Chol content, the mean volume diameter of phytoniosomes was also increased and this effect was significantly detectable in S60/T60 formulations as shown in Table 3 (*P *< 0.05). In a similar investigation, a decreased vesicle size was observed when the HLB value was decreased or the alkyl chain length was increased (38). In the present study, similar results were observed about S40/T40 and S60/T60 nME. 

*Shape of phytoniosomes*

The optical and scanning electron micrographs of nME illustrated that the formed vesicles were uniform in sizes and nearly spherical in shapes (Figure 1).

*Zeta-potential value*

Theoretically, niosome formation requires addition of amphiphiles to improve vesicles stability and prevent aggregation via electrostatic repulsion. Stearylamine and cetylpyridinium chloride are generally used as charge-inducing agents to impart positive charges, and dicetylphosphate and phosphatidic acid induce negative charges to the vesicular surface (31). This surface charge in vesicles is expressed as zeta-potential value. We have found that empty niosomes of S40/T40 and S60/T60 in the presence of Chol have zeta potential values of 2.96 ± 0.05 to 4.13 ± 0.03 mV. The zeta-potential values of phytoniosomes were found to be high even without the addition of any class of amphiphile (Table 3). This could be attributed to the multi component nature of ME.

*Encapsulation efficiency*

The EE% of the nME was determined by analysis of entrapped extract presented in the niosomal pellets after separation by centrifugation. The ME content in the vesicles was evaluated spectrophotometrically using the Folin-Ciocalteu reagent assay.

The nature and intensity of the interaction of a drug with the lipid bilayer primarily depend on the three-dimensional chemical structure, hydrophilicity, and dipole moment or charge of the substance (28). In general, increasing the hydrocarbon chain length from C12 to C18 and saturating the hydrophobic chain (stearyl vs. oleyl increases the rigidity of the bilayer of the sorbitan ester niosomes. Hence, the encapsulation of poorly soluble compounds or water soluble molecules will be improved (27). The EE% of nME varied from 45.4% to 93.4% as shown in Table 3.

Increasing the amount of Chol from 30 to 50 molar ratios enhances the EE% for the S60/T60. Data in Table 3 reveals that the EE% for nME prepared using S60, was superior to that prepared using S40. S40 has lower phase transition temperature and a shorter saturated alkyl chain (C14) compared to S60 (C16), hence nME prepared with S40/T40 has lower EE%. Similar to the nME prepared with S60, the higher drug EE% and stability were achieved with the lower HLB of the surfactant (39).

*Stability testing*

To evaluate stability of vesicles, storage of phytoniosomes was down at 4 °C, 25 °C with RH of 30%, and 40 °C with RH of 70%. Then, stability was determined by assessing the changes in size, constituent separation, and EE% at 24 h 2 weeks, 1 and 3 months after nME preparation (Table 4). Vesicles size has been increased during storage, specially, in the case of formulations that stored at 40 °C with RH of 70%. Increase in size may be related to the fusion and aggregation of vesicles during storage time. The maximum and minimum of mean size changes, during 3 months storage were for F2 formulation stored at 40 °C with RH of 70%, and F5 formulation stored at 4 °C, respectively (Figure 2). Phytoniosomes stored at 25 °C with RH of 30% and 40 °C with RH of 70% underwent decomposition. Furthermore, separate crystal and particle originated from phytoniosomes constituents also were formed. In agreement with our findings, other studies reported similar results about the instability of vesicles in same conditions (9, 30). The current results showed that nME were stable during 3 months at 4 °C. It was observed that S60/T60 phytoniosomes which were stored at 4 °C showed higher physical stability over other formulations. In consistent with present results, Varshosaz *et al*. showed that ascorbic acid niosomes were stable during 6 months at 4 °C (40). Thus, F5 formulation was considered as an optimal formulation because of high stability and EE% during storage time. 

*Drug release*

The rate of drug release must be determined in order to achieve an optional delivery system with desired release characteristics for formulation. However, the rate of release from niosomal formulations is dependent on several factors including the size of the entrapped molecules, vesicle structure, presence or absence of the charge inducer agents and finally chemical structure of the Chol and non-ionic surfactants (40). 

As illustrated in Figure 3 A, B, ME solution gave a release percentages about 51.97% and 66.16% after 1 h through acetate cellulose membrane, 0.45 µm, and regenerated cellulose membrane, 0.45 µm, respectively, whereas the F5 formulation demonstrated only 10.06% and 6.03% drug release after 1 h. The active constituents released from the ME began to plateau after 3 h in both membrane discussed above, whereas the release from the F5 formulation was continued for 6 h and plateau was observed after 8 h. Figure 3 C revealed that ME released from cellophane dialysis sack 12000 Da could not access plateau after 6 h, but F5 formulation reached plateau after 9 h with maximum 26.73% phytoconstituents release. The release data mathematically were analyzed accordingly to zero order, first-order, Higuchi’s and Peppas equations. The data were best fitted to Peppas equation for ME and F5 formulation released from acetate cellulose and regenerated cellulose membrane 0.45 µm. The average R^2^ values were 1 and 0.828 for ME released from acetate cellulose and regenerated cellulose membrane 0.45 µm, respectively and 0.902 and 0.949 for F5 formulation. The average R^2 ^value of 0.997 was calculated for ME released from cellophane dialysis sack 12000 Da and the data were conform zero order equation, but the F5 formulation R^2 ^value was 0.970 and best fitted to Higuchi’s equation. As shown in Table 5, these results demonstrated prolonged-release characteristics of ME, where phytoniosomes act as a reservoir system for continuous delivery of phytoconstituents. Also the physical stability of F5 formulation during release process from different membranes was assessed. Our results demonstrated that there wasn’t any significant change in vesicular size during release process (Figure 4).

*Evaluation of ME and F5 formulation cytotoxicity effect*

To compare the cytotoxicity of ME and F5 formulation, 3T3cells were incubated with different concentrations of ME and F5 formulation (0.1-7µg/mL) for 24 h. As demonstrated in Figure 5, F5 formulation significantly showed lower toxicity on 3T3 cells at concentration 7(µg/mL)that might be due to ME entrapment and sustained release. Gunes showed that empty niosome composed of nonionic surfactants and Chol had not any toxic effect against treated HeLa and A549 cell lines at concentrations of 0.2-10 mM. Furthermore, cell viabilities were diminished by oleander methanol extract and its niosomal formulation at concentrations of 0.1-0.5 mg/mL (41). Another study displayed that viability of Vero cells did not significantly change by treating with marigold niosome and empty niosome up to concentrations of 0.16 mM (13).

*Antibacterial activity*

The antibacterial activity of ME and F5 formulation was determined against *S. aureus*, *S. epidermidis*, *E. coli*, *M. luteus*, and *B. subtilis*. Current results have shown that all the species were sensitive to ME and F5 formulation and they grew in the presence of empty niosomes. As indicated in Table 6, in some concentrations F5 formulation had higher antibacterial activity that have been shown by lower MICs and higher zone of inhibition compared to ME. According to the MICs, *S. aureus* and *B. subtilis* were more sensitive species. Taheri *et al. *indicated that ME was effective against *S. aureus*, *E. Coli,* and *Vibrio cholera*. Similar to our investigation, they reported that the minimum MIC was against *S. aureus* (0.2 mg/mL) and the maximum one was against *E. coli* (8 mg/mL) (42). As indicated in the previous report, ME showed acceptable antibacterial activity against pathogens and it could be used in both the food industry and for medicinal purpose (43). However, there are some limitations for ME usage due to its poor biopharmaceutical properties, low solubility, and low permeability. Therefore, drug delivery systems such as niosome may be practical through enhancing the ME stability, solubility, and bioavailability (44). The application of ME liposome may alleviate the problem of bacterial resistance shown by Gortzi *et al.* report. Their results showed that liposomal formulation increased the stability and bioavailability of extract and possessed antimicrobial activity (45).

## Conclusion

To enhance therapeutic effects and bioavailability, an enormous number of attempts have been made with regard to development of drug delivery systems based on herbs and their phytoconstituents. nME were prepared and optimized by film hydration method. Multi lamellar vesicles were obtained from the S40/T40 and S60/T60 surfactants in the presence of Chol. The S60: T60: Chol (3:3:4 molar ratio) with 4% ME was the optimal formulation, which was stable at 4 °C more than 3 months. Vesicle dispersion acquired from S60/T60 had higher EE%, approximately 91.5%. Encapsulation of ME in niosome could reduce the release rate and prolong the duration of action. Furthermore, the F5formulation showed lower toxicity effect on 3T3 cells and better antibacterial activity compared with ME.

## References

[1] Sewell RD, Rafieian-Kopaei M (2014). The history and ups and downs of herbal medicine usage. J. Herb. Med. Pharmacol.

[2] Semalty A, Semalty M, Rawat M SM, Franceschi F (2010). Supramolecular phospholipids–polyphenolics interactions: The PHYTOSOME® strategy to improve the bioavailability of phytochemicals. Fitoterapia.

[3] Saraf S (2010). Applications of novel drug delivery system for herbal formulations. Fitoterapia.

[4] Baena-Aristizábal CM, Mora-Huertas CE (2013). Micro, nano and molecular novel delivery systems as carriers for herbal materials. J. Colloid Sci. Biotechnol.

[5] Mahale N, Thakkar P, Mali R, Walunj D, Chaudhari S (2012). Niosomes: novel sustained release nonionic stable vesicular systems—an overview. Adv. Colloid Interface Sci.

[6] Sankhyan A, Pawar P (2012). Recent trends in niosome as vesicular drug delivery system. J. Appl. Pharm. Sci.

[7] Ritwiset A, Krongsuk S, Johns JR (2016). Molecular structure and dynamical properties of niosome bilayers with and without cholesterol incorporation: A molecular dynamics simulation study. Appl. Surf. Sci.

[8] Kumar G P, Rajeshwarrao P (2011). Nonionic surfactant vesicular systems for effective drug delivery—an overview. Acta. Pharm. Sin. B.

[9] Kamble B, Talreja S, Gupta A, Patil D, Pathak D, Moothedath I, Duraiswamy B (2013). Development and biological evaluation of Gymnema sylvestre extract-loaded nonionic surfactant-based niosomes. Nanomed.

[10] Chandel A, Saroha K, Nanda S (2012). Preparation and evaluation of proniosomal gel of neem seed oil. Int. J. Pharm. Sci. Nanotechnol.

[11] Nematollahi MH, Torkzadeh-Mahanai M, Pardakhty A, Ebrahimi Meimand HA, Asadikaram G (2017). Ternary complex of plasmid DNA with NLS-Mu-Mu protein and cationic niosome for biocompatible and efficient gene delivery: a comparative study with protamine and lipofectamine. Art. Cells. Nanomed. Biotechnol.

[12] Hoh C, Boocock D, Marczylo T, Singh R, Berry DP, Dennison AR, Hemingway D, Miller A, West K, Euden S (2006). Pilot study of oral silibinin, a putative chemopreventive agent, in colorectal cancer patients: silibinin levels in plasma, colorectum, and liver and their pharmacodynamic consequences. Clin. Cancer Res.

[13] Un R N, Barlas F B, Yavuz M, Ag Seleci D, Seleci M, Gumus Z P, Guler E, Demir B, Can M, Coskunol H (2015). Phyto-Niosomes: In-vitro Assessment of the Novel Nanovesicles Containing Marigold Extract. Int. J. Polym. Mater. Polym. Biomater.

[14] Kumar K, Rai A (2011). Development and evaluation of proniosome-encapsulated curcumin for transdermal administration. Trop. J. Pharm. Res.

[15] Jin Y, Wen J, Garg S, Liu D, Zhou Y, Teng L, Zhang W (2013). Development of a novel niosomal system for oral delivery of Ginkgo biloba extract. Int. J. nanomed.

[16] Manosroi A, Chutoprapat R, Abe M, Manosroi W, Manosroi J (2012). Anti-aging efficacy of topical formulations containing niosomes entrapped with rice bran bioactive compounds. Pharm. Biol.

[17] Manosroi A, Jantrawut P, Akihisa T, Manosroi W, Manosroi J (2011). In-vitro and in-vivo skin anti-aging evaluation of gel containing niosomes loaded with a semi-purified fraction containing gallic acid from Terminalia chebula galls. Pharm. Biol.

[18] Yeh MI, Huang HC, Liaw JH, Huang MC, Huang KF, Hsu FL (2013). Dermal delivery by niosomes of black tea extract as a sunscreen agent. Int. J. Dermatol.

[19] Mozaffarian A (1996). Dictionary of Iranian Plant Names.

[20] Gerbeth K, Hüsch J, Meins J, Rossi A, Sautebin L, Wiechmann K, Werz O, Skarke C, Barrett JS, Schubert-Zsilavecz M (2012). Myrtucommulone from Myrtus communis: metabolism, permeability, and systemic exposure in rats. Planta Med.

[21] Avicenna (1986). Canon of Medicine.

[22] Aghili Khorasani, Alavi Shirazi M H (2011). Makhzan-AL Advieh.

[23] Sarhadynejad Z, Pardakhty A, Mandegary A, Afsharypuor S, Sharififar F (2017). Physicochemical Characterization, Standardization and In-vitro Determination of Radical Scavenging Activity of Zereshk-e-Saghir, A Traditional Preparation, and Its Ingredients. J. Young Pharm.

[24] Nematollahi MH, Pardakhty A, Torkzadeh-Mahanai M, Mehrabani M, Asadikaram G (2017). Changes in physical and chemical properties of niosome membrane induced by cholesterol: a promising approach for niosome bilayer intervention. RSC. Adv.

[25] Hosseini S F, Zandi M, Rezaei M, Farahmandghavi F (2013). Two-step method for encapsulation of oregano essential oil in chitosan nanoparticles: preparation, characterization and in-vitro release study. Carbohydr. Polym.

[26] Gardeli C, Vassiliki P, Athanasios M, Kibouris T, Komaitis M (2008). Essential oil composition of Pistacia lentiscus L and Myrtus communis L: Evaluation of antioxidant capacity of methanolic extracts. Food Chemist.

[27] Mokhtar M, Sammour O A, Hammad M A, Megrab NA (2008). Effect of some formulation parameters on flurbiprofen encapsulation and release rates of niosomes prepared from proniosomes. Int. J. Pharm.

[28] Hao Y, Zhao F, Li N, Yang Y, Li Ka (2002). Studies on a high encapsulation of colchicine by a niosome system. Int. J. Pharm.

[29] Uchegbu IF, Vyas SP (1998). Non-ionic surfactant based vesicles (niosomes) in drug delivery. Int. J. Pharm.

[30] Varshosaz J, Pardakhty A, Hajhashemi V-i, Najafabadi AR (2003). Development and physical characterization of sorbitan monoester niosomes for insulin oral delivery. Drug deliv.

[31] Junyaprasert V B, Teeranachaideekul V, Supaperm T (2008). Effect of charged and non-ionic membrane additives on physicochemical properties and stability of niosomes. Aaps. Pharmscitech.

[32] Pardakhty A, Varshosaz J, Rouholamini A (2007). In-vitro study of polyoxyethylene alkyl ether niosomes for delivery of insulin. Int. J. Pharm.

[33] Babaee N, Mansourian A, Momen-Heravi F, Moghadamnia A, Momen-Beitollahi J (2010). The efficacy of a paste containing Myrtus communis (Myrtle) in the management of recurrent aphthous stomatitis: a randomized controlled trial. Clin. Oral Invest.

[34] Mahboubi M (2016). Myrtus communis L. and its application in treatment of Recurrent Aphthous Stomatitis. J. Ethnopharmacol.

[35] Camargo Junior FBd, Gaspar LR, Campos PMBGM (2012). Immediate and long-term effects of polysaccharides-based formulations on human skin. Braz. J. Pharm. Sci.

[36] Hao Y-M, Li Ka (2011). Entrapment and release difference resulting from hydrogen bonding interactions in niosome. Int. J. Pharm.

[37] Di Marzio L, Marianecci C, Petrone M, Rinaldi F, Carafa M (2011). Novel pH-sensitive non-ionic surfactant vesicles: comparison between Tween 21 and Tween 20. Colloids Surf. B.

[38] Agarwal S, Bakshi V, Vitta P, Raghuram A, Pandey S, Udupa N (2004). Effect of cholesterol content and surfactant HLB on vesicle properties of niosomes. Indian J. Pharm. Sci.

[39] Guinedi A S, Mortada N D, Mansour S, Hathout RM (2005). Preparation and evaluation of reverse-phase evaporation and multilamellar niosomes as ophthalmic carriers of acetazolamide. Int. J. Pharm.

[40] Varshosaz J, Taymouri S, Pardakhty A, Asadi-Shekaari M, Babaee A (2014). Niosomes of ascorbic acid and α-tocopherol in the cerebral ischemia-reperfusion model in male rats. BioMed. Res. Int.

[41] Gunes A, Guler E, Un R N, Demir B, Barlas FB, Yavuz M, Coskunol H, Timur S (2017). Niosomes of Nerium oleander extracts: In-vitro assessment of bioactive nanovesicular structures. J. Drug Deliv. Sci. Technol.

[42] Taheri A, Seyfan A, Jalalinezhad S, Nasery F (2013). Antibacterial effect of Myrtus communis hydro-alcoholic extract on pathogenic bacteria. Zahedan J. Res. Med. Sci.

[43] Aleksic V, Knezevic P (2014). Antimicrobial and antioxidative activity of extracts and essential oils of Myrtus communis L. Microbiol. Res.

[44] Raeiszadeh M, Pardakhty A, Sharififar F, Farsinejad A, Mehrabani M, Hosseini-nave H, Mehrabani M (2018). Development, physicochemical characterization, and antimicrobial evaluation of niosomal myrtle essential oil. Res. Pharm. Sci.

[45] Gortzi O, Lalas S, Chinou I, Tsaknis J (2008). Reevaluation of bioactivity and antioxidant activity of Myrtus communis extract before and after encapsulation in liposomes. Eur. Food Res. Technol.

